# Prognostic impact of left ventricular mass change in patients with ST-elevation myocardial infarction

**DOI:** 10.1097/MD.0000000000009748

**Published:** 2018-01-26

**Authors:** Jin-Sun Park, Jeoung-Sook Shin, You-Hong Lee, Kyoung-Woo Seo, Byoung-Joo Choi, So-Yeon Choi, Myeong-Ho Yoon, Gyo-Seung Hwang, Seung-Jea Tahk, Joon-Han Shin

**Affiliations:** Department of Cardiology, Ajou University School of Medicine, Suwon, Korea.

**Keywords:** left ventricular hypertrophy, myocardial infarction, prognosis

## Abstract

Prognostic significance between progression of left ventricular hypertrophy (LVH) and clinical outcomes in patients with ST-elevation myocardial infarction (STEMI) is uncertain. The objective of this study was to investigate prognostic impact of progression of LV mass index (LVMI) in patients with STEMI.

We analyzed the data and clinical outcomes of patients with STEMI who received successful coronary intervention. A total of 200 patients who had echocardiographic follow-up between 12 and 36 months were finally enrolled. According to change in LVMI compared to baseline LVMI, patients were classified into progression group and nonprogression group. Progression of LVMI was defined when increment of LMVI was greater than 10% compared to baseline LVMI. End points were major adverse cardiac events within 5 years, including death, recurrent MI, target vessel revascularization, and hospitalization due to heart failure.

Progression of LVMI occurred in 55 patients. In the progression group, rate of recurrent MI was higher (13 vs 2%, *P* = .026) and the event-free survival of recurrent MI was significantly worse (log-rank *P* < .001) than that in the nonprogression group. Adjusted hazard ratio of progression of LVMI for recurrent MI was 10.253 (95% confidence intervals 2.019–52.061, *P* = .005).

Increased LVMI was an independent predictor for adverse events, especially for recurrent MI, in patients with STEMI.

## 1 Introduction

Left ventricular hypertrophy (LVH) is closely related to adverse cardiovascular events in various etiologies.^[1–3]^ We have previously reported that LVH is associated with increased rate of adverse clinical outcomes in 30-day survivors after ST-elevation myocardial infarction (STEMI) with successful percutaneous coronary intervention (PCI).^[4]^ In the previous study, we examined the prognostic significance of LVH only at baseline without evaluating the impact of their serial changes. The progression or regression of LVH might affect the clinical outcomes. However, prognostic significance between progression of LVH and clinical outcomes in patients with STEMI has not been established yet. Therefore, the objective of this study was to determine the prognostic impact of progression of LVH in patients with STEMI.

## 2 Methods

### 2.1 Subject population

We consecutively enrolled 30-day survivors after STEMI who underwent successful revascularization. Successful revascularization was defined as thrombolysis in myocardial infarction trial (TIMI) grade 3 flow and < 30% residual stenosis in infarct related artery after primary PCI. Transthoracic echocardiography was performed within 48 hours of primary PCI. We finally enrolled 200 patients (133 males, 56 ± 11 year-old) with echocardiographic follow-up (F/U) between 12 and 36 months after index STEMI. Medical records of all patients were retrospectively reviewed. This study was approved by the Ajou University Hospital Institutional Review Board (approval number: AJIRB-MED-MDB-17–015). We excluded patients from this study if they had history of prior revascularization. We also excluded patients if the LV dysfunction was caused by any of the following: predisposing cardiomyopathy, severe valvular heart disease including symptomatic aortic stenosis, or more than moderate aortic and mitral regurgitation.

### 2.2 Definition of left ventricular mass and its progression

Left ventricular mass (LVM) was calculated according to Devereux's formula^[5]^ using linear measurements derived from two-dimensional echocardiography.^[6]^ 
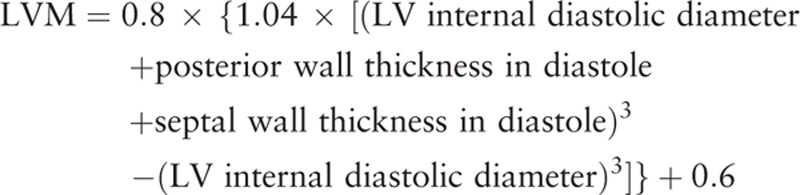


LVM was indexed to body surface area. According to the changes in LVMI compared to baseline LVMI, patients were classified into progression group and nonprogression group. Progression of LVMI was defined when increment of LMVI was greater than 10% compared to baseline LVMI.

### 2.3 Study end-points

End points of the present study were major adverse cardiac events (MACEs) within 5 years, including death, recurrent myocardial infarction (MI), target vessel revascularization (TVR), and hospitalization due to heart failure (HF). Recurrent myocardial infarction was defined according to the universal definition of MI.^[7]^ Target vessel revascularization was defined as clinically indicated percutaneous or surgical revascularization of the index vessel during follow-up. At 5 years after index STEMI, follow-up data were obtained by reviewing medical records and/or telephone interview with patients. To demonstrate a correlation between progression of LVH and long-term clinical outcomes, we enrolled the patients from 2003 to 2009, who were followed-up for more than 5 years.

### 2.4 Statistical analysis

SPSS 13.0 statistical software package (SPSS, Chicago, Illinois) was used for all analyses. Data are shown as mean ± standard deviation for continuous variables or numbers and percentages for categorical variables. Comparisons were conducted by unpaired Student's *t* test for continuous variables or Pearson chi-square test for categorical variables. Event free survival analysis for patients in these groups was performed using the Kaplan–Meier method. Differences between groups were assessed by log-rank test. To assess adjusted relative hazard ratio (HR) of progression of LVMI to the study end points, Cox's proportional hazard model was used with potential variables associated with clinical outcomes. Adjusted covariates for the Cox's proportional hazard model were well-known predictors of MACEs such as age, gender, diabetes mellitus, hypertension, smoking, dyslipidemia, Killip classification, LV ejection fraction (EF), and progression of LVMI. Results of Cox's regression analysis were expressed as adjusted HRs with 95% confidence intervals (CI) for clinical outcomes. Multivariate logistic regression analysis was performed to assess the effect of the presence of progression of LVMI on clinical outcomes. Null hypotheses of no difference were rejected if *P* values were less than .05.

## 3 Results

From 2003 to 2009, a total of 200 patients (164 males, 56 ± 11 year old) were enrolled. Mean value of baseline LVMI of these 200 patients was at 114 ± 30 g/m^2^. Baseline LVMI showed normal distribution. Progression of LVMI occurred in 55 patients (41 males, 58 ± 12 year old). Fifty-five patients (27.5%) were included in the progression group while the remaining 145 patients (72.5%) were included in the nonprogression group.

Baseline clinical characteristics according to the 2 groups are summarized in Table 1. There were no statistical differences in baseline characteristics such as medical history or medical treatments between the 2 groups.

**Table 1:**
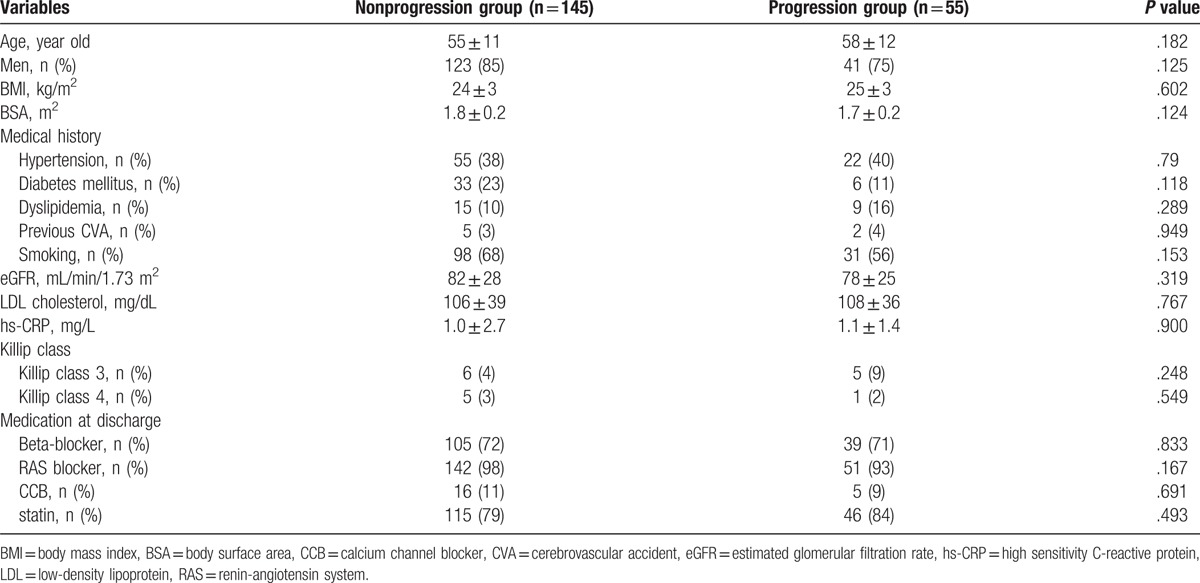
Baseline clinical characteristics.

Patients with three-vessel disease were more common in the progression group (*P* = .02, Table 2) while patient with two-vessel disease were more common in the nonprogression group (*P* = .014). Overall, patients with multivessel disease were not statistically different between the 2 groups. Distributions of culprit lesion and procedural type were also similar between the 2 groups.

**Table 2:**
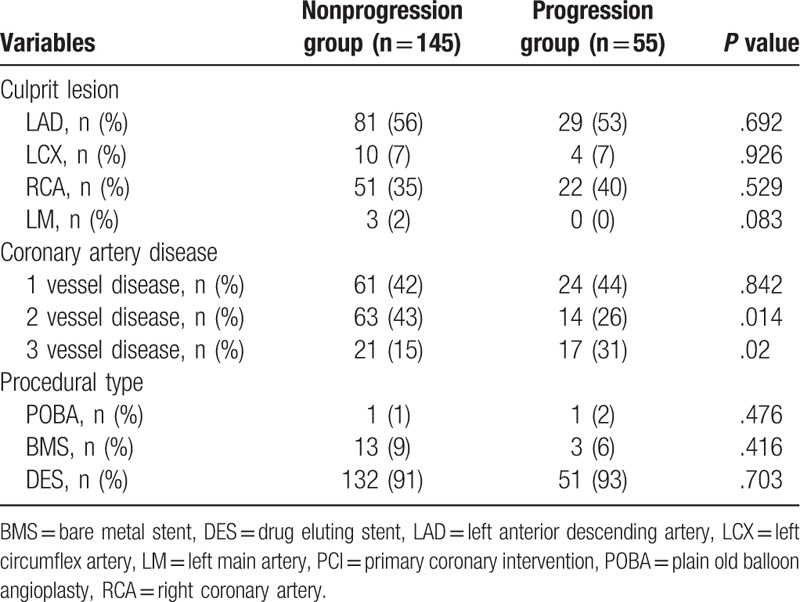
Baseline angiographic characteristics.

Baseline LVMI was significantly lower in the progression group compared to that in the nonprogression group (99 ± 23 vs 120 ± 30 g/m^2^, *P* < .001, Table 3). LV systolic function measured by EF and LV regional function measured by wall motion score index (WMSI) in the progression group was similar to those in the nonprogression LVMI group (EF: 50 ± 9 vs 50 ± 10%, *P* = .775; WMSI: 1.53 ± 0.3 vs 1.54 ± 0.35, *P* = .149). Parameters indicating LV chamber size such as LV end diastolic dimension (LVEDD) and LV end diastolic volume (LVEDV) were also similar between the 2 groups (49 ± 4 vs 51 ± 5 mm, *P* = .457 and 94 ± 24 vs 91 ± 25 mL, *P* = .708, respectively).

**Table 3:**
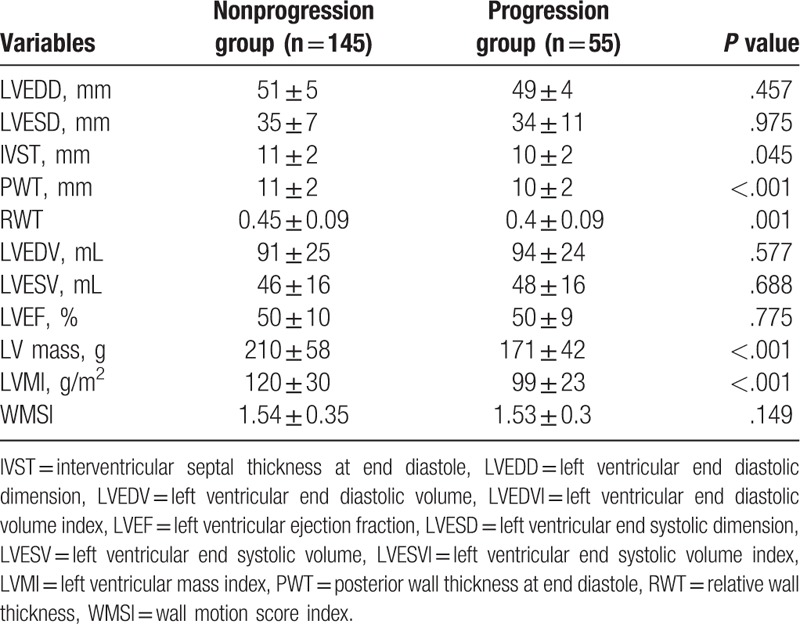
Baseline echocardiographic characteristics.

Mean changes of LVMI in the progression group and those in the nonprogression group were 28 ± 14% and −12 ± 15%, respectively. Follow-up echocardiography showed that LVMI was significantly higher in the progression group than that in the nonprogression group (122 ± 25 vs 104 ± 22 g/m^2^, *P* < .001, Table 4). LV systolic and regional functions were maintained in both groups (EF: 53 ± 12 vs 53 ± 11%, *P* = .966; WMSI: 1.4 ± 0.35 vs 1.39 ± 0.38, *P* = .954). In the progression group, LVEDD and LVEDV, parameters indication LV chamber size, showed progressive LV remodeling compared to those in the nonprogression group (53 ± 5 vs 51 ± 2 mm, *P* = .046; 131 ± 28 vs 113 ± 36 mL, *P = .*035, respectively).

**Table 4:**
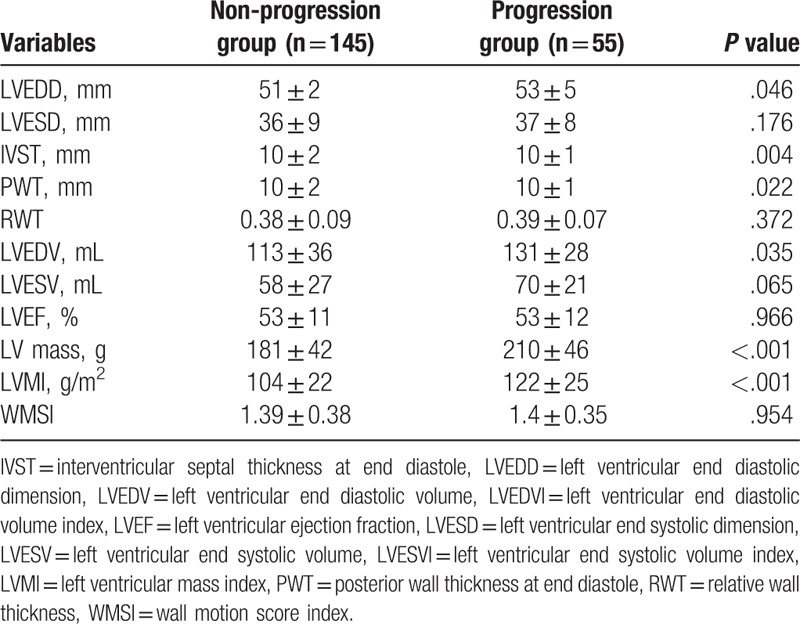
Follow-up echocardiographic characteristics.

**Figure 1:**
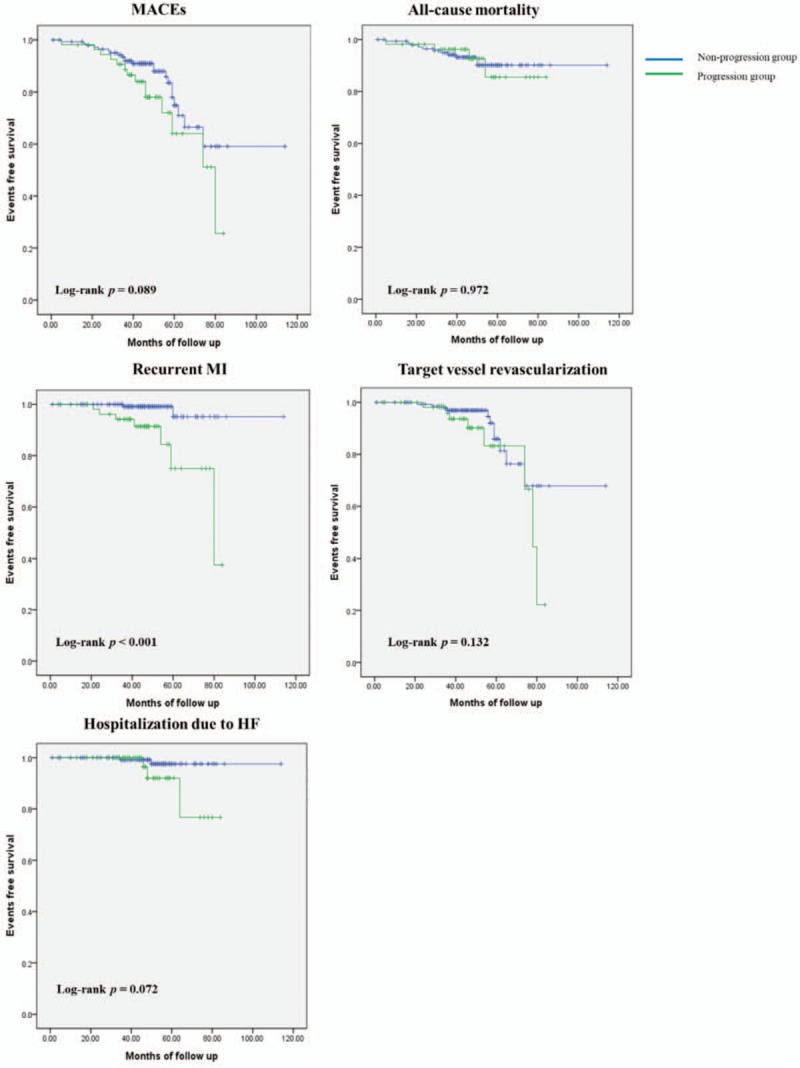
Kaplan–Meier survival curves for free of adverse outcomes in the nonprogression group and the progression group. HF = heart failure, MACEs = major adverse cardiovascular events, MI = myocardial infarction.

Patients were followed-up for 47 ± 16 months after index STEMI. MACEs occurred in 37 patients (19%). Of 200 patients, 15 (8%) died, 10 (5%) experienced recurrent MI, 19 (10%) needed TVR, and 5 (3%) were hospitalized due to HF. Occurrences of MACEs, death, TVR and hospitalization due to HF were similar between the 2 groups (26 vs 16%, 7 vs 8%, 15 vs 8% and 6 vs 1%, respectively). Rate of recurrent MI was higher in the progression group than in the nonprogression group (13 vs 2%, *P = .*026). Event-free survival of recurrent MI was significantly worse in the progression group than in the nonprogression group (log-rank *P* < .001, Fig. 1).

Results of multivariate survival analysis using Cox's regression model are summarized in Table 5. In Cox's proportional hazard model, LVEF (HR 0.956, 95% CI 0.92–0.994, *P = .*023) and progression of LVMI (HR 2.466, 95% CI 1.175–5.174, *P = .*017) were related to MACEs. Age was strongly related to all causes of death (HR 1.1, 95% CI 1.03–1.175, *P = .*004). Adjusted HR of progression of LVMI for recurrent MI was 10.253 (95% CI 2.019–52.061, *P = .*005). LVEF (HR 0.926, 95% CI 0.869–0.987, *P = .*019) and progression of LVMI (HR 3.709, 95% CI 1.257–10.945, *P = .*018) were related to TVR. In a multivariate regression model, progression of LVMI was independently associated with increased risk for recurrent MI (HR 10.833, 95% CI 1.313–89.418, *P = .*027, Table 6).

**Table 5:**
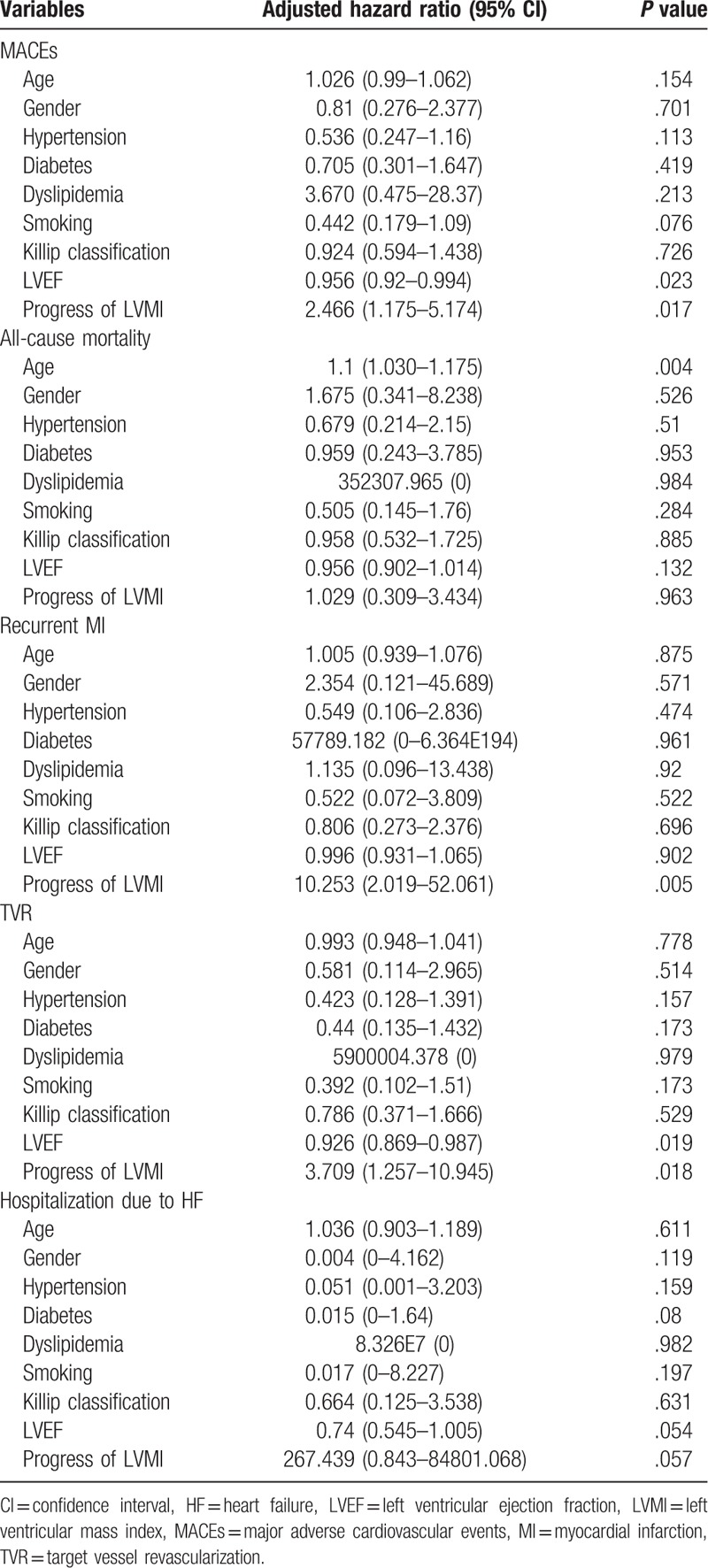
Cox's regression analysis for the adverse outcomes.

**Table 6:**
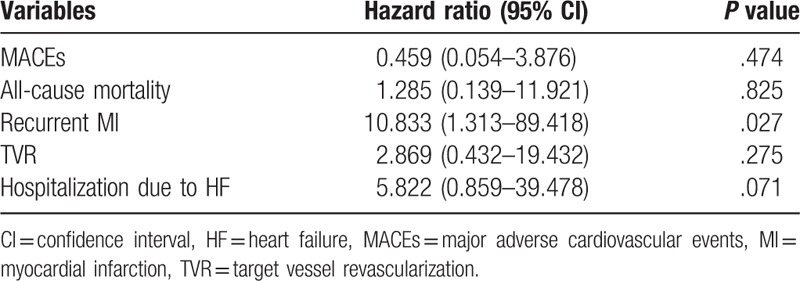
Multivariate logistic regression analysis of the presence of progression of left ventricular mass index for clinical outcomes.

## 4 Discussion

The present study demonstrated that change of LVMI was associated with clinical outcomes in patients with STEMI who received successful PCI. After index STEMI, LV remodeling is divided into an early phase (within 72 hours) and a late phase (beyond 72 hours). Early remodeling is induced by acute loss of myocardium, resulting in abrupt increase in loading conditions.^[8]^ In our previous study, LVH at index STEMI is associated with increased rate of adverse clinical outcomes, especially all-cause mortality. Myocardial structural and functional alterations beyond EF representing LV systolic function might affect adverse clinical outcomes in patients with early remodeling.^[4]^ Late remodeling, including LVH and alterations in ventricular architecture, is an adaptive response that can offset chronically increased hemodynamic load, attenuate progressive dilatation, and stabilize contractile function.^[9]^ The progression of LVMI was related to increased risk for recurrent MI in the present study. In Cox's proportional hazard model, progression of LVMI was related to recurrent MI and TVR. Progression of LVMI might be more closely related to coronary vascular complication.

Myocyte hypertrophy is initiated by activation of neurohormonal system with local tissue renin-angiotensin (RAS) system and myocardial stretch. After MI, decreased cardiac output enhances cathecholamine production by adrenal medulla and sympathetic nerve terminals. It also activates RAS-aldosterone axis.^[8]^ Stimulation of α1 adrenoreceptor by enhanced norepinephrine (NE) release, and angiotensin 1 receptor can induce myocyte hypertrophy via Gqα-dependent pathway which is upregulated in the viable border and scar tissue in post-MI hearts.^[10]^ Both NE and angiotensin II augment endothelin-1 release, which is another stimulus for myocyte hypertrophy.^[11]^ Mechanical myocardial stretch induced by elevated wall stresses sensed by infarcted and noninfarcted myocardium can result in secretion of angiotensin II from cytoplasmic granules and induce myocyte hypertrophy mediated by angiotensin 1 receptor.^[12]^

In atherosclerotic plaque lesions, local RAS system is also activated. Angiotensin II can induce myocyte hypertrophy. It can also generates oxidative stress in vessel wall, resulting in stimulation of vascular thrombosis and inflammation.^[13]^ In vascular smooth muscle cells, exposure to angiotensin II leads to increased levels of plasminogen-activator inhibitor type 1 which can inhibit tissue plasminogen activator and urokinase, and cell adhesion molecules such as vascular cell adhesion molecule-1 and intercellular adhesion molecule-1, resulting in prothrombotic status.^[14]^ In angiotensin II stimulated vascular smooth muscle cells, effects of inflammatory cytokine interleukin-18 are enhanced. This is related to progression of atherosclerosis and restenosis.^[15]^

Pathologic angiotensin II-induced signaling in vascular, endothelial, and cardiac cells can promote vascular thrombosis, neointima formation, and LVH. In the present study, close correlation between progression of LVMI and adverse clinical outcomes, especially recurrent MI, might be affected by pathologic angiotensin-II signaling. Many studies have demonstrated that RAS blocker can prevent angiotensin II-induced LVH and vascular pathology,^[16–18]^ Therefore, RAS blocker might be able to reduce adverse long-term clinical outcomes in patients with progression of LVMI after index STEMI.

This study has several limitations. First, echocardiographic measuring LVM using linear measurements has potential limitations. It is based on the assumption that the LV is represented by a prolate ellipse.^[5]^ Nevertheless, LVM obtained with this method has been well validated and widely used in clinical practice.^[6]^ Although there could be geometrical deformation, we measured LVMI at index STEMI and follow-up using same technical method and analyzed the presence of change in LVMI not the value of LMVI itself. Second, there is no validated definition of progression of LVMI. In the present study, we defined progression of LVMI as an increment of LMVI greater than 10% compared with baseline LVMI. There have been no data demonstrating association between progression of LVMI and clinical outcomes. Further study is needed to define clinically significant progression of LVMI. Third, the present study could not demonstrate a possible benefit of regression of LVMI. A total of 82 patients had LVMI regression in the present study. There was no significant correlation between regression of LVMI and MACEs. It might be due to the relatively small number of our study population. Fourth, levels of angiotensin II and related cytokines were not checked in the present study. The present study logically implied that angiotensin II had a pathological role in the progression group. To prove this, further studies might be needed to evaluate levels of angiotensin II and related cytokines. Finally, we could not fully evaluate the impact of serial changes of LVMI. Since the present study was retrospective and annual echocardiographic follow-up was not recommended after index STEMI in current guidelines, we enrolled patients with echocardiographic follow-up (F/U) between 12 and 36 months after index STEMI. We could not know the effect of rate of progression in LVMI.

In summary, increased LVMI was found to be an independent predictor for adverse events, especially for recurrent MI in patients with STEMI who received successful coronary intervention. Therefore, maintenance and dose-adjustment of proper medical treatment including RAS blocker should be considered in patients with progression of LVMI after index STEMI.
